# Simultaneous observation of higher-order non-classicalities based on experimental photocount moments and probabilities

**DOI:** 10.1038/s41598-019-45215-x

**Published:** 2019-06-20

**Authors:** Jan Peřina, Ondřej Haderka, Václav Michálek

**Affiliations:** 1 Joint Laboratory of Optics of Palacký University and Institute of Physics of AS CR, Faculty of Science, Palacký University, 17. listopadu 12, 77146 Olomouc, Czech Republic; 2 Institute of Physics of Academy of Sciences of the Czech Republic, Joint Laboratory of Optics of Palacký University and Institute of Physics of AS CR, 17. listopadu 50a, 772 07 Olomouc, Czech Republic

**Keywords:** Quantum optics, Nonlinear optics, Single photons and quantum effects

## Abstract

Using a sub-Poissonian optical field generated from a weak twin beam by photon-number resolving post-selection we have simultaneously observed higher-order non-classicalities in photocount moments (sub-Poissonian statistics) and probabilities (witnessed by the Klyshko inequalities). Up to the seventh-order non-classicalities in photocount moments simultaneously with up to the eleventh-order non-classicalities in photocount probabilities have been experimentally observed. Non-classicality counting parameters of different orders as experimental counterparts of the theoretical Lee non-classicality depth have been suggested to quantify and also mutually compare the robustness of these non-classicalities against the noise.

## Introduction

The formulation of quantum theory of coherence^1,2^ has revealed the existence of special states of light that cannot be described in the framework of classical statistical theory of coherence^3^. These states, that are called nonclassical, immediately attracted attention of experimentalists who began their long-lasting and very successful investigation of such states^4^. Three different kinds of nonclassical states were identified in this endeavor: sub-Poissonian states with reduced intensity (photon-number) fluctuations, squeezed states with reduced phase fluctuations and anti-bunched light with unusual temporal correlations. All three kinds of states were experimentally observed and their properties were analyzed. Moreover, many other highly quantum states have been theoretically suggested and experimentally observed during the last decades^5^ (e.g., non-Gaussian states^6^, sub-binomial states^7^). Whereas highly squeezed states^8^ are nowadays routinely generated in monolithic nonlinear cavities (squeezing better than 12 dB^9^, with the potential to improve the detection of gravitational waves) and strongly anti-bunched light^4^ coming from individual emitters is detected, only weakly sub-Poissonian light has been observed for a long time.

Different techniques for the generation of sub-Poissonian light were applied using resonance fluorescence^10^ (Fano factor $F=\langle {({\rm{\Delta }}n)}^{2}\rangle /\langle n\rangle \approx 0.998$), Franck–Hertz experiment^11^ ($F\approx 0.99$), high-efficiency light-emitting diodes^12^ ($F\approx 0.96$), feed-forward action on the beam^13^, second-subharmonic generation^14^ and excited atoms passing through micro-cavities^15,16^. Also the experimental displaced single-photon states exhibited sub-Poissonian photocount statistics for smaller displacements^17^. Considerable improvement was reached when a post-selection scheme based on photon-number-resolving detectors and exploiting either the continuous signal and idler fields from optical parametric oscillators^18,19^ or pulsed weak twin beams were applied^20–24^. Recently, also collective emissions from small clusters of single-photon emitters were analyzed as promising sources of sub-Poissonian light^25^.

More detailed investigations revealed that the observation of non-classicality of such fields is not restricted to the behavior of second-order photocount moments^26^: Also higher-order photocount and photon-number moments^27,28^ can behave nonclassically. In such cases the observed fields exhibit higher-order non-classicalities^27,29–32^. These higher-order moments are immediately available in various experiments aimed at exploring higher-order correlations in photon fields^33–36^. Nonclassical behavior of higher-order moments was observed for the third-^25^, fourth-^17,35^, fifth-^37^ and even fourteenth-order^38^ photocount moments in different scenarios. On the other hand, also another type of higher-order non-classicalities based on non-classicality inequalities written directly for photocount and photon-number probabilities (elements of the photocount and photon-number distributions) has been recently introduced^39,40^ and experimentally investigated^37^. Both types of non-classicalities are complementary in the sense that one of them ‘scans’ the space of quantum states spanned by photocount (photon-number) moments whereas the other operates directly in the space of photocount (photon-number) distributions.

As it has been demonstrated in ref.^37^ for sub-Poissonian fields generated by photon-number-resolving post-selection from a weak twin beam both types of non-classicalities can be observed simultaneously. Here, we extend the results of ref.^37^ in two directions. First, the improved stability of our experimental setup allows us to report on the simultaneous observation of the seventh-order non-classicality expressed in photocount moments and eleventh-order non-classicality detected in photocount probabilities. Second, we introduce non-classicality counting parameters of different orders that are derived directly from the photocount (photon-number) probabilities or moments. Contrary to the traditionally-used Lee non-classicality depth based on the integrated-intensity (normally-ordered) moments and applied, e.g., in ref.^37^, these parameters are defined also for the non-classicalities written in probabilities. This then allows, among others, mutual comparison of both types of non-classicalities with respect to their robustness against the noise. Also, their evaluation for the case of non-classicalities based on photocount (photon-number) moments does not require the use of commutation relations. This is especially important for multi-mode optical fields.

The fact that both types of higher-order non-classicalities can be identified and quantified already in the experimental photocount distributions (histograms) is principally important as it allows to avoid possible ‘distortions’ of non-classical properties of the detected fields caused by their reconstruction. Excluding the death-time effect and its spatial variant in photon detection^41^, attenuation in detection (described by quantum detection efficiency), the detection of additional noise photons and cross-talk effects only degrade nonclassical properties of the detected optical fields. Subsequent reconstruction of the optical field then compensates for these effects which may result in artifacts in identification of the optical-field non-classicality. For this reason, special non-classicality inequalities valid directly for the photocount distributions measured by multiple on-off detectors (‘click-counting’ statistics) were even derived^42^.

The paper is organized as follows. In section Higher-order non-classicalities and their quantification, we define higher-order non-classicalities of two types and the corresponding non-classicality counting parameters. Properties of these counting parameters are theoretically investigated in section Non-classicality counting parameters *τ* for specific groups of states. In section Experimental higher-order non-classicalities in photocount moments and probabilities the non-classicality parameters are applied to an experimentally generated sub-Poissonian field with about 11 photons on average. Analysis of nonclassical properties of the corresponding photon-number distribution is contained in section Higher-order non-classicalities in the reconstructed field. Section Conclusions summarizes the results.

## Higher-Order Non-Classicalities and Their Quantification

Non-classicality is defined in general as a property of a state endowed with non-positive Glauber-Sudarshan phase-space quasi-distribution. It has been shown in refs^4,28,37^ that a state with a *k*th photocount (photon-number) moment $\langle {c}^{k}\rangle$ smaller than that of a classical optical field with a Poissonian photocount (photon-number) statistics ${\langle {c}^{k}\rangle }_{{\rm{Pois}}}$ (in a coherent state) with the same mean photocount (photon) number $\langle c\rangle ={\langle c\rangle }_{{\rm{Pois}}}$ is nonclassical. This brings us to the following definition of a *k*th-order non-classicality observed in photocount (photon-number) moments^37^ that refers to the *k*th-order sub-Poissonian statistics:

1 $${r}_{c}^{(k)}=\frac{\langle {c}^{k}\rangle }{{\langle {c}^{k}\rangle }_{{\rm{Pois}}}}-1 < 0,\,k=2,3,\ldots .$$

We note that the states exhibiting second-order as well as higher-order non-classicalities defined by inequality (1) form a subset inside the set of all nonclassical states fulfilling the above general definition.

Theoretical analysis of the measured photocount and also reconstructed photon-number distributions reveals a complementary way how to identify another type of higher-order non-classicalities^39^. It is based directly upon the elements *f*(*c*) [*p*(*n*)] of the photocount [photon-number] distributions that, for any classical field, have to obey the following inequalities^37,39^:

2 $$k!f(k)f{(0)}^{k-1}-f{(1)}^{k}\ge 0,\,k=2,3,\ldots .$$

We note that the Poissonian distribution *f*(*c*) gives equality in Eq. (2). In parallel to Eq. (1), we may define the following coefficient ${\tilde{r}}_{c}^{(k)}$ that identifies a *k*th-order non-classicality observed directly in the (modified) photocount (photon-number) distributions^39^ [$\tilde{f}(k)=k!f(k)/f(0)$]:

3 $${\tilde{r}}_{c}^{(k)}=\frac{\tilde{f}(k)}{\tilde{f}{(1)}^{k}}-1 < 0,\,k=2,3,\ldots .$$

At the theoretical level, the Lee non-classicality depth^43^ is standardly used to quantify robustness of nonclassical features of light. However, the determination of the Lee non-classicality depth is based upon the (normally-ordered) integrated intensity moments that have to be derived from the usual experimental photocount (photon-number) moments relying on the commutation relations between the field’s creation and annihilation operators. To avoid this derivation that does not have to be apparent for multi-mode fields, we suggest here a non-classicality counting parameter *τ* for quantifying the robustness of non-classicality. It is based upon considering directly the photocount (photon-number) distribution of the analyzed field. According to the definition, we superimpose an independent noise thermal field with varying mean photocount (photon) number *τ* to the analyzed field. When the noise mean photocount (photon) number *τ* increases, non-classicality of the composed field characterized by the overall photocount (photon-number) distribution *f*_comp_,

4 $${f}_{{\rm{comp}}}(c;\tau )=\sum _{c^{\prime} =0}^{c}\,f(c-c^{\prime} )\frac{{\tau }^{c^{\prime} }}{{(1+\tau )}^{c^{\prime} +1}},$$

weakens. The threshold values of *τ* at which the coefficients ${r}_{c}^{(k)}$ in Eq. (1) and ${\tilde{r}}_{c}^{(k)}$ in Eq. (3) vanish can then be used as non-classicality quantifiers with respect to a given criterion. The greater the non-classicality counting parameter *τ* is, the more robust the non-classicality is. The values of *τ* are found in the range from 0 to $\infty$. However, the parameter *τ* cannot be successfully applied to highly nonclassical states that remain nonclassical even for $\tau \to \infty$ as shown in the following section.

## Non-Classicality Counting Parameters *τ* for Specific Groups of States

To elucidate the behavior of non-classicality counting parameters ${\tau }^{(k)}$ and ${\tilde{\tau }}^{(k)}$ defined above, we determine the non-classicality coefficients ${r}_{c}^{(k)}$ and ${\tilde{r}}_{c}^{(k)}$ defined in Eqs (1) and (3), respectively, for two groups of states: mixtures composed of the vacuum state $|{\rm{vac}}\rangle$ and one-photon Fock state $\mathrm{|1}\rangle$ and Fock states $|N\rangle$ with varying photon number *N*. We assume ideal photon-number detection in which the photocount distribution *f*(*c*) coincides with the photon-number distribution *p*(*n*).

Considering the first group the states are described by the statistical operator $\hat{\rho }$ given as

5 $$\hat{\rho }(\alpha )=(1-\alpha )|{\rm{vac}}\rangle \langle {\rm{vac}}|+\alpha |1\rangle \langle 1|$$

and $\alpha \in \langle 0,1\rangle$ is a real parameter. They have simple photon-number distributions $p(n)$ that give the following photocount distributions $f(c)$:

6 $$f(0)=1-\alpha ,\,f(1)=\alpha ,\,f(k)=0,\,k=2,\ldots .$$

Photocount distribution ${f}_{{\rm{comp}}}(c,\tau )$ defined according to Eq. (4) is derived for the distribution $f(c)$ in Eq. (6) in the form:

7 $$\begin{array}{rcl}{f}_{{\rm{comp}}}(0;\tau ) & = & (1-\alpha )\frac{1}{1+\tau },\\ {f}_{{\rm{comp}}}(k;\tau ) & = & (1-\alpha )\frac{{\tau }^{k}}{{(1+\tau )}^{k+1}}+\alpha \frac{{\tau }^{k-1}}{{(1+\tau )}^{k}},\,k=1,\ldots .\end{array}$$

Photocount moments $\langle {c}^{k}\rangle$ of a noise thermal distribution as well as of the reference Poissonian distribution needed when determining coefficients ${r}_{c}^{(k)}$ can conveniently be derived invoking the relation between photocount (photon-number) and integrated-intensity (*W*_*c*_) moments^3^:

8 $$\langle {c}^{k}\rangle =\sum _{l=1}^{k}\,{S}_{kl}\langle {W}_{c}^{l}\rangle ;$$

symbol *S*_*kl*_ stands for the Stirling numbers of the second kind. We have for the noise thermal distribution with mean integrated intensity $\langle {W}_{c}\rangle$:

9 $$\langle {W}_{c}^{l}\rangle =l!\,{\langle {W}_{c}\rangle }^{l}.$$

On the other hand, the following integrated-intensity moments characterize the Poissonian field with mean integrated intensity $\langle {W}_{c}\rangle$:

10 $$\langle {W}_{c}^{l}\rangle ={\langle {W}_{c}\rangle }^{l}.$$

Applying formula (9) to the composed photocount distribution ${f}_{{\rm{comp}}}(c,\tau )$ in Eq. (7), we arrive at the following first photocount moments:

11 $$\begin{array}{rcl}\langle c\rangle  & = & \alpha +\tau ,\\ \langle {c}^{2}\rangle  & = & (\alpha +\tau )(1+2\tau ).\end{array}$$

Formula (10) then allows us to arrive at the second-order non-classicality coefficient ${r}_{c}^{\mathrm{(2)}}$:

12 $${r}_{c}^{(2)}(\tau ;\alpha )=\frac{-\,{\alpha }^{2}+{\tau }^{2}}{(\alpha +\tau )(\alpha +\tau +1)}.$$

Considering ${r}_{c}^{(2)}=0$ in Eq. (12) the non-classicality counting parameter ${\tau }_{c}^{\mathrm{(2)}}$ is derived as

13 $${\tau }_{c}^{(2)}(\alpha )=\alpha .$$

According to Eq. (13), the greater the fraction *α* of the one-photon Fock state in the analyzed state is, the greater amount *τ* of thermal noise is needed to conceal the non-classicality, and thus the more nonclassical the state is.

For the composed photocount distribution ${f}_{{\rm{comp}}}(c)$ with the elements given in Eq. (7) the coefficients ${\tilde{r}}_{c}^{(k)}$ from Eq. (3) are derived as follows

14 $${\tilde{r}}_{c}^{(k)}(\tau ;\alpha )=\frac{k!\,({\tau ^{\prime} }^{k}+\alpha ^{\prime} {\tau ^{\prime} }^{(k-1)})}{{(\alpha ^{\prime} +\tau ^{\prime} )}^{k}}-1,$$

$\tau ^{\prime} =\tau$/$(1+\tau )$ and $\alpha ^{\prime} =\alpha$/$(1-\alpha )$. Considering the condition ${\tilde{r}}_{c}^{(2)}=0$ in Eq. (14) we arrive at the corresponding non-classicality counting parameter ${\tilde{\tau }}_{c}^{\mathrm{(2)}}$ for $\alpha \le 1$/2:

15 $${\tilde{\tau }}_{c}^{(2)}(\alpha )=\frac{\alpha }{1-2\alpha }.$$

On the other hand, the coefficient ${\tilde{r}}_{c}^{\mathrm{(2)}}$ remains negative (and hence nonclassical) for $\alpha  > 1$/2 even for $\tau \to {\rm{\infty }}$. This means that the non-classicality counting parameter ${\mathop{\tau }\limits^{ \sim }}_{c}^{(2)}$ is not sensitive enough to quantify robustness of these highly nonclassical states.

We note that the analytical formulas for the non-classicality counting parameters ${\tau }_{c}^{\mathrm{(3)}}$ and ${\tilde{\tau }}_{c}^{\mathrm{(3)}}$ reveal the inequalities ${\tau }_{c}^{\mathrm{(3)}}\le {\tau }_{c}^{\mathrm{(2)}}$ and ${\tilde{\tau }}_{c}^{(3)}\le {\tilde{\tau }}_{c}^{(2)}$.

Performing similar calculations for an *N*-photon Fock state $|N\rangle$ with statistical operator ${\hat{\rho }}_{N}$ ($N\ge 1$),

16 $${\hat{\rho }}_{N}=|N\rangle \langle N|,$$

we arrive at the following formula for the second-order non-classicality coefficient ${r}_{c}^{\mathrm{(2)}}$:

17 $${r}_{c}^{(2)}(\tau ;N)=\frac{{\tau }^{2}-N}{{(\tau +N)}^{2}+\tau +N}.$$

Assuming ${r}_{c}^{(2)}=0$ in Eq. (17) the non-classicality counting parameter ${\tau }_{c}^{(2)}(N)$ is derived as

18 $${\tau }_{c}^{(2)}(N)=\sqrt{N}.$$

Thus, it exceeds one for $N > 1$. We note that the coefficients ${\tilde{r}}_{c}^{(k)}(\tau ;N)$ are not defined for these states and, similarly as above, we also have ${\tau }_{c}^{(3)}\le {\tau }_{c}^{(2)}$.

## Experimental Higher-Order Non-Classicalities in Photocount Moments and Probabilities

Now we analyze the performance of the introduced non-classicality counting parameters ${r}_{c}^{(k)}$ and ${\tilde{r}}_{c}^{(k)}$ in case of an experimental photocount histogram *f*(*c*) belonging to a sub-Poissonian field obtained by photon-number-resolving post-selection from a weak twin beam. We show that though the non-classicality counting parameters are not applicable for highly nonclassical states they successfully quantify the non-classicality of real experimental fields.

**Figure 1:**
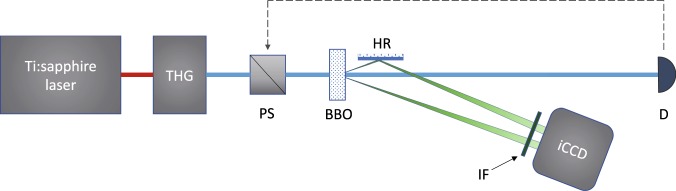
Scheme of the performed experiment: A twin beam is emitted in a BaB_2_O_4_ (BBO) crystal pumped by the third harmonics (THG) of a femtosecond Ti:sapphire laser. The signal field and the idler field (after reflection on mirror HR), after filtering by bandpass interference filter IF, are detected by iCCD camera. Intensity of the pump beam stabilized by power stabilizer PS is monitored by detector D.

A twin beam that was used for the post-selection was emitted in non-collinear geometry in a 5-mm-long type-I BaB_2_O_4_ crystal pumped by the third harmonics (280 nm) of a femtosecond cavity dumped Ti:sapphire laser (pulse duration 150 fs, central wavelength 840 nm) (for the setup, see Fig. 1). The signal field as well as the idler field were detected by the photocathode of an intensified CCD (iCCD) camera Andor DH334-18U-63 whose detection efficiency $\eta =0.220\pm 0.005$ and dark-count rate $DM=0.040\pm 0.005$ electrons per frame was determined in an independent measurement^22,26^. We note that also intensified CMOS cameras^44,45^ and electron-multiplied CCD cameras^46^ were used as photon-number-resolving detectors in similar tasks. Both the signal and idler beams were monitored by $M=6500$ neighbor pixels that provided photon-number resolution^41,47^. In the experiment the nearly-frequency-degenerate signal and idler fields at the wavelength of 560 nm were filtered by a 14-nm-wide bandpass interference filter. Intensity of the pump beam was actively stabilized via a motorized half-wave plate followed by a polarizer based on the information about intensity from a detector. The analyzed idler field was obtained by considering only the experimental realizations in which exactly *c*_s_ = 5 signal photocounts were observed. Out of the overall 1.2 × 10^6^ repetitions of the measurement, 8.49% of them provided the analyzed sub-Poissonian field. For the generated twin beam with averaged photon-pair number equal to 8.800 ± 0.003 and negligible amount of noise (≈0.1), the mean photocount number $\langle {c}_{{\rm{i}}}\rangle$ of the post-selected idler field equaled 2.770 ± 0.003 and its Fano factor ${F}_{c,{\rm{i}}}=0.94\pm 0.01$ confirmed the sub-Poissonian statistics.

**Figure 2:**
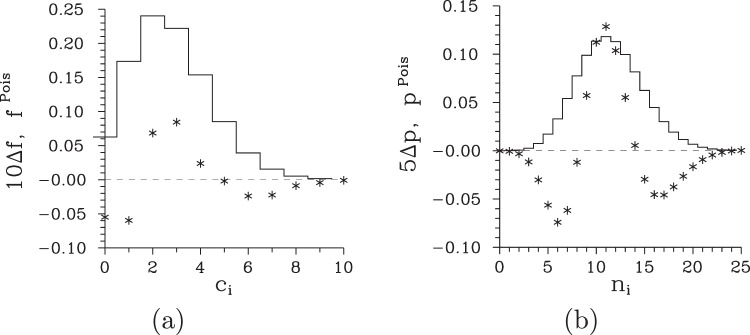
(**a**,**b**) Difference Δ*f* [Δ*p*] between the photocount histogram *f* [photon-number distribution *p*] and its Poissonian counterpart *f*^Pois^ [*p*^Pois^] is plotted (*) for the signal photocount number ${c}_{s}=5$. For comparison, Poissonian histogram *f*^Pois^ [Poissonian photon-number distribution *p*^Pois^] is plotted by a solid curve.

**Figure 3:**
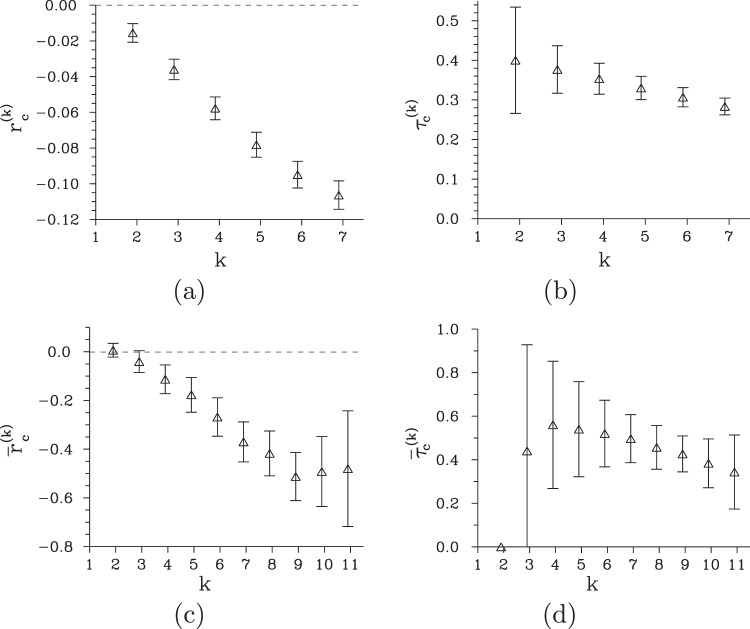
(**a**,**c**) Non-classicality identifiers ${r}_{c}^{(k)}$ [${\tilde{r}}_{c}^{(k)}$] and the corresponding (**b**,**d**) non-classicality depths ${\tau }_{c}^{(k)}$ [${\tilde{\tau }}_{c}^{(k)}$] of the post-selected idler field for the experimental photocount histogram *f*(*c*_i_) as they depend on the order *k* of non-classicality.

Declination Δ*f* of the obtained experimental photocount histogram *f*(*c*_i_) from the corresponding Poissonian distribution plotted in Fig. 2(a) clearly shows narrowing of the experimental histogram $f({c}_{{\rm{i}}})$ with respect to the Poissonian reference. This narrowing gives sub-Poissonian character to the field, as already indicated by the Fano factor ${F}_{c,{\rm{i}}}$ lower than 1. Application of the higher-order non-classicality identifiers ${r}_{c}^{(k)}$ and ${\tilde{r}}_{c}^{(k)}$ based on photocount moments and probabilities and defined in Eqs (1) and (3), respectively, reveals nonclassical properties of this field. According to the graphs in Fig. 3(a,c), the field exhibits up to the seventh-order non-classicality in the coefficient ${r}_{c}^{(k)}$ and, simultaneously, up to the eleventh-order non-classicality in the ‘complemenatary’ coefficient ${\tilde{r}}_{c}^{(k)}$. The observation of even higher-order non-classicalities suffers from the experimental noise. Increase of the number of measurement realizations would be needed to reach them experimentally. We note, that fields exhibiting up to the fourteenth-order non-classicality in photocount moments [${r}_{c}^{(k)}$] were reported in^38^ using a similar post-selection scheme with a superconducting photon-number-resolving detector with higher efficiency.

The higher-order non-classicality counting parameters ${\tau }_{c}^{(k)}$ and ${\tilde{\tau }}_{c}^{(k)}$, that allow for the quantification of non-classicality resistance against the noise, are plotted in Fig. 3(b,d) for the analyzed higher-order non-classicalities. According to the graph in Fig. 3(b), the resistance of higher-order non-classicality based on photocount moments (${r}_{c}^{(k)}$) decreases with the order parameter *k*. On the other hand, the higher-order non-classicality counting parameter ${\tilde{\tau }}_{c}^{(k)}$ derived from photocount probabilities reveals non-classicality on the right-hand side from the maximum in the photocount distribution *f*(*c*_i_) [see Fig. 2(a)]: The greater the photocount number *k* is the more prone the non-classicality identifier ${\tilde{r}}_{c}^{(k)}$ to the noise is. Mutual comparison of the values of non-classicality counting parameters ${\tau }_{c}^{(k)}$ and ${\tilde{\tau }}_{c}^{(k)}$ plotted in Fig. 3(b,d), respectively, reveals better resistance of the parameters ${\tilde{\tau }}_{c}^{(k)}$ derived from photocount probabilities against the noise.

## Higher-Order Non-Classicalities in The Reconstructed Field

The higher-order non-classicality identifiers ${r}_{n}^{(k)}$ and ${\tilde{r}}_{n}^{(k)}$ and the accompanying non-classicality counting parameters ${\tau }_{n}^{(k)}$ and ${\tilde{\tau }}_{n}^{(k)}$ can also be applied to the reconstructed photon-number distribution *p*(*n*_i_) to judge its non-classicality. Here, we apply the method of maximum likelihood^48^ to arrive at the photon-number distribution *p*(*n*_i_) of the post-selected idler field^22^. According to this method, a photon-number distribution *p*(*n*_i_) is found as a steady state of the following iteration procedure that uses the experimental photocount histogram *f*(*c*_i_)^41^:

19 $${p}^{(l+1)}({n}_{i})={p}^{(l)}({n}_{i})\,\sum _{{c}_{i}}\,\frac{f({c}_{i})T({c}_{i},{n}_{i})}{{\sum }_{{n^{\prime} }_{i}}\,T({c}_{i},{n^{\prime} }_{i}){p}^{(l)}({n^{\prime} }_{i})},\,l=0,1,\ldots .$$

In Eq. (19), the positive-valued operator measure *T* appropriate for the used iCCD camera with *M* active pixels, detection efficiency $\eta$ and dark-count rate per pixel *D* is determined along the formula^41^:

20 $$T(c,n)=(\begin{array}{c}M\\ c\end{array}){(1-D)}^{M}{(1-\eta )}^{n}{(-1)}^{c}\,\sum _{l=0}^{c}\,(\begin{array}{c}c\\ l\end{array})\frac{{(-1)}^{l}}{{(1-D)}^{l}}{(1+\frac{l}{M}\frac{\eta }{1-\eta })}^{n}.$$

**Figure 4:**
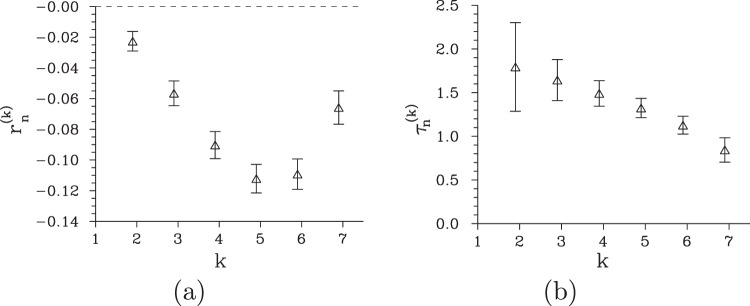
(**a**) Non-classicality identifiers ${r}_{n}^{(k)}$ and (**b**) the corresponding non-classicality depths ${\tau }_{n}^{(k)}$ of the post-selected idler field for the reconstructed photon-number distribution *p*(*n*_i_) as they depend on the order *k* of non-classicality.

The reconstruction method when applied to the experimental histogram *f*(*c*_i_) gives us a field with mean photon number $\langle {n}_{{\rm{i}}}\rangle =11.44\pm 0.01$ and the Fano factor ${F}_{n,{\rm{i}}}=0.72\pm 0.04$. Thus, the reconstruction not only preserved the sub-Poissonian character of the field, it considerably improved its Fano factor *F*_*n*,i_ compared to that appropriate for the photocount histogram. Narrowing of the obtained photon-number distribution *p*(*n*_i_) with respect to its Poissonian counterpart is shown in the graph of Fig. 2(b). The analysis of the reconstructed photon-number distribution *p*(*n*_i_) from the point of view of higher-order non-classicality identifiers ${r}_{n}^{(k)}$ expressed in photon-number moments reveals up to the seventh-order non-classicality [see the graph in Fig. 4(a)], similarly as in the case of photocount histogram *f*(*c*_i_). However, the comparison of the corresponding non-classicality counting parameters ${\tau }_{n}^{(k)}$ plotted in Fig. 4(b) with those determined for photocount histogram *f*(*c*_i_) [see the graph in Fig. 3(b)] reveals better resistance of the former parameters against the noise. For example, whereas around 0.4 noise photocounts conceal the second-order non-classicality ${r}_{c}^{\mathrm{(2)}}$, around 1.8 photons are needed to suppress the second-order non-classicality ${r}_{n}^{\mathrm{(2)}}$. The improved resistance of the non-classicality of the reconstructed field with respect to the noise originates in the increase of the field intensity during the reconstruction. On the other hand, it holds also here that the greater the order *k* of non-classicality the more prone the non-classicality against the noise, as documented in the graph in Fig. 4(b). Unfortunately, the reconstruction that ‘amplifies’ the field roughly four-times considerably broadens the photon-number distribution *p*(*n*_i_) which results in the loss of higher-order non-classicalities ${\tilde{r}}_{n}^{(k)}$ expressed in photon-number probabilities. This stemms from the fact that these probabilities are considerably smaller than those in the original photocount histogram and so the ability to distinguish such non-classicalities is lost in the noise.

## Conclusions

We have analyzed in parallel higher-order non-classicalities defined in terms of photocount or photon-number moments (sub-Poissonian statistics) and probabilities (using the Klyshko inequalities). We have introduced higher-order non-classicality counting parameters for both types of non-classicalities. We have elucidated their general performance considering two types of nonclassical states including the highly nonclassical ones. We have analyzed their performance on an experimental sub-Poissonian field with around 11 photons on average generated from a weak twin beam with around 8.8 mean photon pairs per pulse under the condition of 5 detected signal-beam photocounts. For this field, we have observed up to the seventh-order non-classicality in photocount moments simultaneously with up to the eleventh-order non-classicality in photocount probabilities. The seventh-order non-classicality in photon-number moments has been reached also for the reconstructed photon-number distribution. The introduced higher-order non-classicality counting parameters have shown that, with the increasing non-classicality order, the non-classicality becomes more prone to the noise. Also the non-classicality counting parameters determined for photocount probabilities are more resistant against the noise compared to their counterparts based on photocount moments when weak fields are analyzed.

## Data Availability

All data generated and analyzed during this study are included in this published article.
